# The current role of sodium-glucose cotransporter 2 inhibitors in type 2 diabetes mellitus management

**DOI:** 10.1186/s12933-022-01512-w

**Published:** 2022-05-25

**Authors:** Bo Xu, Shaoqian Li, Bo Kang, Jiecan Zhou

**Affiliations:** 1 The First Affiliated Hospital, Pharmacy Department, Hengyang Medical School, University of South China, Hengyang, 421001 Hunan China; 2 The First Affiliated Hospital, Hengyang Clinical Pharmacology Research Center, Hengyang Medical School, University of South China, Hengyang, 421001 Hunan China; 3 The First Affiliated Hospital, Hengyang Key Laboratory of Clinical Pharmacology, Hengyang Medical School, University of South China, Hengyang, 421001 Hunan China; 4 School of Pharmaceutical Science, Hengyang Medical School, University of South China, Hengyang, 421001 Hunan China

**Keywords:** Sodium-glucose cotransporter 2 inhibitor, Heart failure, Type 2 diabetes mellitus, Cardiovascular disease, Diabetic kidney disease

## Abstract

Type 2 diabetes mellitus (T2DM) is a chronic, complex metabolic disease characterized by chronic hyperglycemia causing from insufficient insulin signaling because of insulin resistance or defective insulin secretion, and may induce severe complications and premature death. Sodium-glucose cotransporter-2 (SGLT2) inhibitors are oral drugs used to reduce hyperglycemia in patients with T2DM, including empagliflozin, ertugliflozin, dapagliflozin and canagliflozin. The primary objective of this article is to examine the clinical benefit, safety, and tolerability of the four SGLT2 inhibitors approved by the US FDA. SGLT2 inhibitors increase urinary glucose excretion via inhibiting SGLT2 to decrease renal reabsorption of filtered glucose and reduce the renal threshold for glucose. Rather than stimulating insulin release, SGLT2 inhibitors improve β-cell function by improving glucotoxicity, as well as reduce insulin resistance and increase insulin sensitivity. Early clinical trials have confirmed the beneficial effects of SGLT2 in T2DM with acceptable safety and excellent tolerability. In recent years, SGLT2 inhibitors has been successively approved by the FDA to decrease cardiovascular death and decrease the risk of stroke and cardiac attack in T2DM adults who have been diagnosed with cardiovascular disease, treating heart failure (HF) with reduced ejection fraction and HF with preserved ejection fraction, and treat diabetic kidney disease (DKD), decrease the risk of hospitalization for HF in T2DM and DKD patients. SGLT2 inhibitors are expected to be an effective treatment for T2DM patients with non alcoholic fatty liver disease. SGLT2 inhibitors have a similar safety profile to placebo or other active control groups, with major adverse events such as Ketoacidosis or hypotension and genital or urinary tract infections.

## Introduction

Diabetes is a major public health problem, affecting approximately 10% of the population [1]. Type 2 diabetes mellitus (T2DM) is a chronic and complex metabolic disease [1–3]. T2DM is characterized by chronic hyperglycemia due to insufficient insulin signaling due to defective insulin secretion and insulin resistance, and it is also related to increased endogenous glucose production (EGP) or glucagon [4]. T2DM patients have a two- to three-fold increased risk of cardiovascular disease, which is further amplified in the presence of chronic renal impairment [3]. In addition to atherosclerotic cardiovascular disease, patients with T2DM have increased risk of developing diabetic kidney disease (DKD) and heart failure (HF) [3, 5, 6].

Hospitalizations for major diabetic complications including myocardial infarction and stroke are increasing [7]. Drug therapy is aimed at preventing microvascular complications, comprising end-stage kidney disease (ESKD) and blindness [1]. As the understanding of the underlying pathophysiological deficits continues to evolve, the therapy options for T2DM have multiplied [6]. The initial approach recommended by the American Association of Clinical Endocrinologists (AACE)/American College of Endocrinology and the American Diabetes Association (ADA) includes lifestyle changes and monotherapy, preferably metformin [6]. Drugs currently commonly used to control blood glucose such as insulin, glucagon-like peptide 1 (GLP1) receptor agonists, sulfonylureas, thiazolidinediones, dipeptidyl peptidase-4 (DPP-4) inhibitors, SGLT2 inhibitors [6]. These drugs have achieved significant clinical success.

Sodium-glucose cotransporter 2 (SGLT2) inhibitors increase urinary glucose excretion via inhibiting glucose reabsorption in the renal proximal tubules [8]. SGLT2 inhibitors can be combined with diet and exercise. They are available as monotherapy or in combination with some antidiabetic drugs, such as metformin [9].

SGLT2 inhibitors reduce glycated hemoglobin (HbA1c) without raising hypoglycemia risk, also they can lose weight and improve hyperuricemia, blood lipids and blood pressure [8]. Current evidence suggest that SGLT2 inhibitors improve renal and cardiovascular outcomes in T2DM patients, especially those with previous cardiovascular events, chronic kidney disease (CKD) or HF [8]. Similarly, several clinical trials using GLP1 receptor agonists and SGLT2 inhibitors have reported data showing cardiovascular benefits and delays in DKD progression [1, 5, 10].

SGLT2 inhibitors have good tolerability, with common adverse events such as genitourinary tract infection, diabetic ketoacidosis (DKA), or hypotension [8, 11–14].

Until now, empagliflozin has been approved by the FDA for T2DM, to reduce cardiovascular death in adults with T2DM, treat adults with HF with reduced ejection fraction (HFrEF) and adults with HF and preserved ejection fraction. Canagliflozin is approved for T2DM and DKD, and reduces the risk of heart attack, cardiovascular death and stroke in adults with T2DM and established cardiovascular disease, and reduces the risk of hospitalization for heart failure (HHF) in T2DM and DKD patients. Dapagliflozin is indicated for T2DM, reduce the risk of HHF, for the treatment of HF in patients with HFrEF, and for CKD patients with or without the risk of T2DM progression. Ertugliflozin is approved for T2DM [15–18].

The main purpose of this review is to examine the clinical role, clinical advantages, safety and tolerability of SGLT2 inhibitors.

## Essential effects of SGLT2 inhibitors

SGLT2 (Table 1) is the major transporter responsible for the reabsorption of glucose from the glomerular filtrate back into the circulation, is characterized by low affinity, high volume, as well as is predominantly expressed in the proximal tubules [19, 20]. By inhibiting SGLT2, SGLT2 inhibitors lower renal reabsorption of filtered glucose and reduce the renal threshold for glucose, thereby increasing urinary glucose excretion [19, 21–27]. This reduces HbA1c by approximately 0.6–1.0% [8, 28]. SGLT2 inhibitors increase sodium delivery to distal tubules through blocking SGLT2-dependent glucose and sodium reabsorption, which is thought to increase tube-ball feedback and decrease intraglomerular pressure. This may affect a variety of physiological functions, comprising reducing cardiac preload and afterload and downregulating sympathetic nerve activity [4, 19, 21–28]. Metabolism is shifted to gluconeogenesis and ketosis, which are thought to have protective effects on the heart and kidneys [28]. SGLT2 inhibitors can reduce glucotoxicity in renal tubular cells by reducing mitochondrial dysfunction and inflammation, and also reduce renal hypoxia by reducing tubular energy and oxygen demand [28].

**Table 1:** Clinical effects and risks of SGLT2 inhibitors

Essential clinical role
1. Effect on glycemic
A.The value of HbA1c level reduced approximately 0.7–1.0%
B.Lower fasting plasma glucose, about 1.2 mmol/L
C.Improved beta cell function
D.Improve glucotoxicity
E.Improved insulin sensitivity
F.Increased endogenous glucose production
G.Reduced insulin resistance
2. Effects on lipids
A.Increased HDL, LDL and apolipoprotein
B.Does not alter the concentration of LDL-C, but decreases small, dense LDL-C and increases large buoyant LDL-C
C.Increased total cholesterol
D.Lower triglyceride levels
E.Lead to increased adiponectin
3. Weight
A.Decreased waist circumference, subcutaneous adipose tissue, and visceral adipose tissue
B.Increased glucagon secretion
C.Weight loss of about 2–4 kg
4. Cardiovascular effects
A.Reduced systolic blood pressure
B.Improve inflammation and oxidative stress
C.Improve vascular function
D.Reduce cardiac preload
E.Decreased left ventricular mass index
F.Decreased NT-proBNP concentration
G.Improve natriuresis
H.Reduces pathological cardiomyocyte stiffness
I.Lead to osmotic diuresis
J.Increase hematocrit
K.Lung fluid volume improved
L.Improves diastolic function
M.Improve cardiac remodeling
N.Reduce ischemia–reperfusion injury
5. Effects on fibrosis markers
A.Decreased Mac-2 binding protein
B.FIB-4 index decreased
C.Significantly lower NAFIC scores
6. Liver function improvement
A.Reduced fatty liver index
B.Decreased serum alanine aminotransferase, aspartate aminotransferase and gamma-glutamyltransferase levels
C.Proton density fat fraction decreased
7. Effects on the kidneys
A.Reducing vascular volume
B.Reduce blood uric acid levels
C.Reduced proteinuria
D.Stabilize eGFR
E.Improve urinary albumin/creatinine ratio
F.Reduced kidney disease progression
G.Improve natriuresis
8. SGLT2 inhibitors may reduce bone density
9. Effects on inflammatory factors
A.Decreses soluble dipeptidyl peptidase levels
B.Decreases ICAM-1, VCAM-1, TNF-α and IL-6
10. Oxidative stress
A.Reduce H_2_O_2_, GSH, lipid peroxide
B.Prevent PKGIα oxidation
11. Effect on anemia
A.Decrease hepcidin levels
B.Improve erythropoiesis
C.Increase hemoglobin levels

Clinically established and indeterminate ameliorated events with SGLT2 inhibitors and possible risks following administration	
Established ameliorated events	Risks
Cardiovascular: heart failure and hospitalization for heart failure, cardiovascular death, atrial fibrillation	Hypoglycemia
	Hypotension
All-cause mortality	Sarcopenia
Anemia	Acute kidney injury
Nonalcoholic fatty liver	Diabetic ketoacidosis
Renal protection: diabetic kidney disease, chronic kidney disease, end-stage renal disease, kidney failure, kidney death	Genital or urinary tract infection
	Dehydration
	Hypovolemia
Indeterminate ameliorated events	Osmotic diuresis
Nonfatal myocardial infarction	Amputation
Non-fatal stroke	Fracture

The expression of *SLC5A2* was higher in islets of non-T2DM patients than in islets of T2DM individuals or normal islets exposed to chronic hyperglycemia, with lower glucagon gene expression [4]. Glucagon secretion can be achieved by inhibiting *SLC5A2* by siRNA-induced gene silencing or by inhibiting SGLT2 activation through K_ATP_ channels by dapagliflozin [4]. Glucagon secretion and hepatic gluconeogenesis were improved in normal mice treated with dapagliflozin, and fasting-induced hypoglycemia was limited [4, 29]. The effect of SGLT1 on glucagon secretion depends on glucose transport, not glucose metabolism. Canagliflozin inhibits glucagon secretion by inhibiting SGLT1 in alpha cells [29].

SGLT2 inhibitors can induce transforms in IL-6, adiponectin, and serum leptin and improve adipose tissue function, which have favorable effects on insulin sensitivity and cardiovascular disease risk [30, 31]. SGLT2 inhibitors also increase HDL, LDL levels and reduce triglyceride levels [32, 33]. SGLT2 inhibitors are able to obviously reduce systolic blood pressure (SBP) and lead to weight loss (Table 2). Additionally, a meta-analysis of 58 studies reported favorable effects of SGLT2 inhibitors on HbA1c levels (mean difference (MD) from placebo − 0.66%; active comparator − 0.06%). SGLT2 inhibitors decreased body weight (− 1.80 kg) and SBP (− 4.45 mm Hg) compared to other drugs [34].

**Table 2:** Effects of SGLT-2 on change from baseline HbA1c (%), FPG (mmol/L), body weight (kg) and SBP (mmHg) in patients with T2DM, changes from baseline results of randomized controlled trials

Study^a^	Patients	Drugs	Number	FPG	HbA1c	Weight loss	SBP
CANTATA-SU [99]	T2DM patients with 7.0–9.5% HbA1c	Canagliflozin 100 mg	483	− 1.35	− 0.82	− 3.7	− 3.3
		Canagliflozin 300 mg	485	− 1.52	− 0.93	− 4.0	− 4.6
		Glimepiride	482	− 1.02	− 0.81	0.7	0.2
Lavalle-González et al. [100]	Patients with T2DM had inadequate glycaemic control	Sitagliptin	366	− 1.0	− 0.73	− 1.3	− 0.7
		Canagliflozin 100 mg	368	− 1.5	− 0.73	− 3.8	− 3.5
		Canagliflozin 300 mg	367	− 2.0	− 0.88	− 4.2	− 4.7
		Placebo/sitagliptin	183		− 0.17^c^	− 1.2^c^	
Bruce Bode [101]	Patients with 7.0% ≤ HbA1c ≤ 10.0%	Placebo	237	0.4	− 0.03	− 0.1	1.1
		Canagliflozin 100 mg	241	− 1.0	− 0.60	− 2.2	− 3.5
		Canagliflozin 300 mg	236	− 1.1	− 0.73	− 2.8	− 6.8
Michael Roden et al. [108]	Adults had no treatment in the previous 12 weeks	Placebo	228	0.65	0.08	− 0.33	− 0.3
		Empagliflozin 10 mg	224	− 1.08	− 0.66	− 2.26	− 2.9
		Empagliflozin 25 mg	224	− 1.36	− 0.78	− 2.48	− 3.7
		Sitagliptin	223	− 0.38	− 0.66	0.18	0.5
Ferrannini [110]	Patients with T2DM	Empagliflozin 10 mg	80	− 30^b^	− 0.34	− 2.2	0.1
		Empagliflozin 25 mg	88	− 28^b^	− 0.47	− 2.6	− 1.7
		Metformin	56	− 26^b^	− 0.56	− 1.3	2.0
Duration-8 [121]	Patients with T2DM inadequately controlled by metformin	Exenatide plus dapagliflozin	228	− 3.66	− 2.0	− 3.55	− 4.3
		Exenatide	227	− 2.54	− 1.6	− 1.56	− 1.2
		Dapagliflozin	230	− 2.73	− 1.4	− 2.22	− 1.8
Weber et al. [89]	Patients with inadequately controlled T2DM and hypertension	Placebo	224	0.2	− 0.02	− 0.59	− 7.62
		Dapagliflozin	225	− 1.0	− 0.63	− 1.44	− 11.90
VERTIS Asia [123]	Patients with T2DM inadequately controlled on metformin	Placebo	167	− 6.7^b^	− 0.2	− 1.2	0.2
		Ertugliflozin 5 mg	170	− 37.1^b^	− 1.0	− 3.0	− 5.1
		Ertugliflozin 15 mg	169	− 34.5^b^	− 0.9	− 3.2	− 3.9
VERTIS MET [126]	Adults with T2DM inadequately controlled on metformin	Placebo/glimepiride	209	− 0.6	− 0.6	− 0.18	0.05
		Ertugliflozin 5 mg	207	− 1.0	− 0.6	− 3.77	− 3.61
		Ertugliflozin 15 mg	205	− 1.6	− 0.9	− 3.63	− 3.13
VERTIS FACTORIAL [127]	Patients with HbA1c ≥ 7.5% and ≤ 11.0%	Ertugliflozin 5 mg	250	− 28.7	− 1.0	− 2.4	− 2.7
		Ertugliflozin 15 mg	248	− 30.8	− 0.9	− 3.2	− 1.6
		Sitagliptin	247	− 15.2	− 0.8	− 0.1	− 0.2
		Ertugliflozin 5 mg/sitagliptin	243	− 39.3	− 1.4	− 2.4	− 2.3
		Ertugliflozin 15 mg/sitagliptin	244	− 41.8	− 1.4	− 2.8	− 2.2

*FPG* fasting plasma glucose, *SBP* systolic blood pressure, *T2DM *type 2 diabetes mellitus, *HbA1c *glycated haemoglobin

^a^Outcome data for all trials correspond to data at the longest dosing time in each trial

^b^The unit here is mg/dL

^c^Week 26 results here

### Effect on beta cells

Rather than stimulating insulin release, SGLT2 inhibitors improve β-cell function by improving glucotoxicity and reducing β-cell workload [8, 20]. SGLT2 inhibitor-induced glucosuria improved beta-cell function and insulin sensitivity, decreased tissue glucose disposal and increased EGP following administration, thereby reducing fasting and postprandial glycemic [35]. Eight weeks of treatment with empagliflozin restored hypothalamic insulin sensitivity, possibly contributing to the beneficial effects of SGLT2 inhibitors [36].

### Regulate blood lipids

A single-center, prospective trial determined that SGLT2 inhibitors increased HDL-C and apolipoprotein AI compared with sitagliptin. SGLT2 inhibitors did not change the concentration of LDL-C, while reduced small, dense LDL-C by 20% and increased large buoyant LDL-C by 18%. SGLT2 inhibitors increased HDL2-C by 18% without affecting HDL3-C [37]. A meta-analysis of 15 studies indicated no association between SGLT2 inhibitors and dyslipidemia risk [ratio risk = 1.13]. It was significantly associated with a 0.15 mmol/L increase in total cholesterol, a 0.12 mmol/L increase in LDL, a 0.07 mmol/L increase in HDL, and a reduction in triglycerides of 0.12 mmol/L compared to controls [33].

### Effect on liver

Nonalcoholic fatty liver disease (NAFLD), which often coexists with T2DM, is the most prevalent chronic liver disease worldwide [38–40]. Clinically, SGLT2 inhibitors have shown benefit for NAFLD.

In the E-LIFT trial, SGLT2 inhibitors were obviously better at decreasing liver fat than standard therapy [MRI-Proton Density Fat Fraction (PDFF) difference − 4.0%; P < 0.0001]. MRI-PDFF was notably lower in the SGLT2 inhibitor group (P < 0.0001) compared with baseline. Two groups displayed a significant difference in serum alanine aminotransferase (ALT) levels (P = 0.005) [38]. Two meta-analyses results showed that SGLT-2 inhibitors significantly reduced serum ALT, aspartate aminotransferase and γ-glutamyltransferase levels compared with controls, and the absolute percentage of liver fat content based on magnetic resonance technology, and body composition, glycemic control, lipid parameters, and markers of inflammation were significantly improved, and there was a trend for improvement in markers of fibrosis [41, 42].

In NAFLD patients, dapagliflozin significantly reduced the fatty liver index (P < 0.01) compared with pioglitazone, and changes in the fatty liver index were significantly positively correlated with changes in insulin (P < 0.01) and HbA1c (P = 0.03) levels [43].

### Cardiovascular benefits

The investigators concluded that the cardioprotective effects of SGLT2 inhibitors can be attributed to (Table 1): blood pressure control, increased plasma erythrocytes levels, decreased inflammation and oxidative stress, decreased uric acid, prevention of ischemia/reperfusion injury and improved cardiac and vascular function [8, 30, 32, 44]. The effects of SGLT2 inhibitors in the early stages, in addition to direct improvements in peripheral endothelial function, are responsible for the clinical benefit found in the Cardiovascular Outcomes Trial [45]. SGLT2 inhibitors lead to acute and chronic reductions in SBP, reductions in vasoconstrictors and increases in vasodilators. These changes may contribute to its antihypertensive effect and its benefit in congestive HF [46]. Early pathogenesis of human diabetic cardiomyopathy is associated with JunD/PPAR-γ overexpression and lipid accumulation after heart transplantation in diabetic patients. This phenomenon was decreased by therapy with SGLT2 inhibitors that act directly on the heart of diabetic patients [47].

EMPA-HEART CardioLink-6 was designed to determine whether empagliflozin causes decreased left ventricular mass in coronary artery disease patients with T2DM [48]. For patients assigned to empagliflozin and placebo, mean body surface area-related left ventricular mass regressions over 6 months was 2.6 g/m^2^ and 0.01 g/m^2^, respectively (P = 0.01) [48]. SGLT2 inhibitors was related to obviously lower extracellular volume, left ventricular mass index, and indexed extracellular compartment volume, which may be partly responsible for the beneficial cardiovascular outcomes [48, 49].

An analysis of the results of the CANVAS program (Canagliflozin Cardiovascular Assessment Study) indicated that patients with a previous cardiovascular event had higher absolute rates of cardiovascular, renal and death outcomes compared with participants without a previous cardiovascular event. Canagliflozin reduced cardiovascular outcomes overall [50]. Data from the DIVERSITY-CVR trial demonstrated that SGLT2 inhibitors were obviously more effective than sitagliptin in improving cardiometabolic risk factors [51]. Results of an exploratory analysis of EMPA-REG OUTCOME indicated that changes in hemoglobin and hematocrit mediated 48.9% and 51.8%, respectively, of the effect of empagliflozin vs. placebo on the risk of cardiovascular death, while HbA1c, fasting plasma glucose (FPG) and uric acid were less regulated (29.3%) [52]. Ertugliflozin was noninferior to placebo in terms of major adverse cardiovascular events (MACE) in patients with atherosclerotic cardiovascular disease and T2DM [11].

In a retrospective cohort study, patients administered SGLT2 inhibitors experienced a lower risk of developing HHF [hazard ratio (HR) = 0.86], all-cause mortality (HR = 0.85), and stroke (HR = 0.86) compared with DPP-4 inhibitors [53]. A multi-database retrospective cohort study also identified that SGLT2 inhibitors were related to a reduced risk of HF (0.43), cardiovascular death (0.60), MACE (HR = 0.76) and myocardial infarction (0.82) compared with DPP-4 inhibitors. Canagliflozin (HR = 0.79), dapagliflozin (0.73), and empagliflozin (0.77) had similar benefits for MACE [54].

Multiple meta-analyses have reported that SGLT2 inhibitors were related to fewer cardiovascular events, especially HHF and cardiovascular mortality. However, for non-fatal stroke and myocardial infarction differ [12, 55–60]. SGLT2 inhibitors reduced MACE by 11% (P = 0.0014), with benefit only in atherosclerotic cardiovascular disease patients (HR = 0.86) [61]. A meta-analysis of 764 studies (n = 421,346) reported that SGLT-2 inhibitors reduced non-fatal myocardial infarction, and cardiovascular death. Importantly, SGLT-2 inhibitors decreased HHF more than GLP-1 [55]. Results of a network meta-analysis of 453 studies showed that oral SGLT-2 inhibitors decreased HHF in patients at increased cardiovascular risk who had prior metformin-based therapy [12]. Another meta-analysis included data from 57 trials (n = 33,385) and 6 regulatory submissions (n = 37,525), providing data on seven SGLT2 inhibitors [60]. SGLT2 inhibitors prevented the risk of MACE (relative risk 0.84; P = 0.006), all-cause death (0.71; P < 0.0001), HF (0.65; P = 0.002), and cardiovascular death (0.63; P < 0.0001). There was no significant effect on angina (0.95; P = 0.70) or non-fatal myocardial infarction (0.88; P = 0.18) [60].

#### Benefits of SGLT2 inhibitors in HF, HFrEF and HHF

SGLT2 inhibitors reduce left ventricular volume in T2DM or prediabetic patients with HFrEF. Favorable reverse left ventricular remodeling may be a mechanism by which SGLT2 reduces HHF and mortality in patients with HFrEF [62]. The partial of patients receiving SGLT2 inhibitors improved significantly in lung fluid volume [63]. Elevated concentrations of N-terminal pre-B-type natriuretic peptide (NT-proBNP) are related to HF diagnosis and prediction of cardiovascular risk [64]. A significant proportion of patients in the CANVAS trial had increased NT-proBNP values. Canagliflozin reduced NT-proBNP concentrations compared with placebo. However, the reduction in NT-proBNP explains only a small fraction of the benefit of canagliflozin on HF events [64]. A meta-analysis showed that SGLT2 inhibitors was related to obviously improved diastolic function and NT-proBNP levels in T2DM patients with or without chronic HF, while it did not significantly affect the structural parameters of the heart based on body surface area. Left ventricular ejection fraction levels improved only in patients with HFrEF [65]. SGLT2 inhibitors reduce inflammation and oxidative stress in HF with preserved ejection fraction, improve NO-sGC-cGMP-cascade and PKGIα activity by reducing PKGIα oxidation and aggregation, thereby reducing pathological cardiomyocyte stiffness [66]. Results from the large multinational study CVD-REAL demonstrated that SGLT-2 inhibitors were related to lower HHF and mortality compared with other antidiabetic agents [67].

Some randomized controlled trials have also conformed the benefit of SGLT2 inhibitors in HF or HHF (Table 3) [13, 68–72]. In the CANVAS program, canagliflozin significantly reduced severe HF or HHF (P = 0.003) and HHF (P = 0.002) compared with placebo. Canagliflozin had a greater benefit of cardiovascular death or HHF in patients with a prior history of HF (HR 0.61 vs. 0.87; P = 0.021) compared patients without HF at baseline [72]. Empagliflozin was related to a 35 percent decrease in the relative risk of HHF compared with placebo in EMPA-REG OUTCOME [69]. In EMPEROR-Reduced, the incidence of HHF was obviously lower in empagliflozin compared to placebo in adults with or without T2DM with HFrEF (HR = 0.69). And the overall number of people with HHF was less (21.0% vs. 30.0; P < 0.001) [68]. In DAPA-HF, the risk of HHF was lower in the dapagliflozin group for patients with (12.8% vs. 16.2%; P = 0.017) or without (7.2% vs. 11.2%; P < 0.001) T2DM [71]. Approximately 12.3% patients with HFrEF accompany by COPD, and these patients were at higher risk for the primary outcome. The benefit of dapagliflozin on prespecified outcomes was consistent regardless of COPD [73]. Ertugliflozin was also able to decrease the risk of HHF to some extent compared with placebo in the VERTIS CV trial [11, 74]. The risk reduction for first HHF was similar in patients with ejection fraction ≤ 45% or preserved ejection fraction > 45% or unknown [74].

**Table 3:** The clinical cardiovascular events occurrence (%) of SGLT2 inhibitors, results from major randomized controlled trials

Study	Patients	Duration	Drugs	Number	MACE	HHF	CV death	Nonfatal MI	Stroke^a^
CREDENCE [70]	Patients with T2DM and albuminuric CKD	2.62 years	Canagliflozin	2202	12.4	4.0	5.0		
			Placebo	2199	16.4	6.4	6.4		
			P value			< 0.001	0.05		
EMPA-REG OUTCOME [69]	T2DM patients	3.1 years	Empagliflozin	4687	10.5	2.7	3.7	4.5	3.2
			Placebo	2333	12.1	4.1	5.9	5.2	2.6
			P value		< 0.001	0.002	< 0.001	0.22	0.16
EMPEROR-Reduced [68]	Patients with HF and an EF of 40% or less	16 months	Empagliflozin	1863	19.4	13.2	10.0		
			Placebo	1867	24.7	18.3	10.8		
			P value		< 0.001				
DECLARE–TIMI 58 [13]	T2DM patients and/or atherosclerotic CV disease	4.2 years	Dapagliflozin	8582	8.8	2.5	2.9	4.6^b^	2.7
			Placebo	8578	9.4	3.3	2.9	5.1^b^	2.7
			P value		0.17				
DAPA-HF [79]	Patients with HF and an EF of 40% or less	18.2 months	Dapagliflozin	2373	16.3	9.7	9.6		
			Placebo	2371	21.2	13.4	11.5		
			P value		< 0.001				
DAPA-CKD [122]	Patients with Chronic Kidney Disease	2.4 years	Dapagliflozin	2152	4.6		3.0		
			Placebo	2152	6.4		3.7		
			P value		0.009				
VERTIS CV [11]	T2DM patients with atherosclerotic CV disease	3.5 years	Ertugliflozin	5499	11.9	2.5	6.2	5.6	2.9
			Placebo	2747	11.9	3.6	6.7	5.4	2.8
			P value		< 0.001				
CANVAS Program^c^ [50]	T2DM patients with high CV risk		Canagliflozin	5795	26.9	5.5	11.6	9.7	7.1
			Placebo	4347	31.5	8.7	12.8	11.6	8.4
			P value^d^		0.5980	0.2359	0.9387	0.9777	0.4978

*MACE* major adverse cardiovascular events, *HHF *hospitalized for heart failure, *CV *cardiovascular, MI myocardial infarction, *T2DM* type 2 diabetes mellitus, *CKD *chronic kidney disease, *EF* ejection fraction

^a^Stroke includes ischemic stroke or non-fatal stroke

^b^Here only for MI

^c^Rates of various cardiovascular events were replaced with rates per 1000 persons per year

^d^P-value for homogeneity between CANVAS and CANVAS-R

Several meta-analyses have indicated that SGLT-2 inhibitors reduce HHF and are related to lower HF compared with placebo or active control group [12, 55, 56, 59, 61, 75–77]. SGLT-2 inhibitors were notably related to a lower incidence of HF events (HR = 0.62) [75]. SGLT2 inhibitors decreased the risk of cardiovascular death or HHF by 23% (P < 0.0001) and HHF by 31% (P < 0.0001), similar benefits were seen in patients with or without a history of HF or atherosclerotic cardiovascular disease [61]. Canagliflozin (HR = 0.69, 0.68, 0.67, 0.58) or empagliflozin (HR = 0.70, 0.69, 0.68, 0.59) notably decreased HHF compared with duraglutide, exenatide, lixisenatide, and subcutaneous injection [56]. In a real-world meta-analysis OBSERVE-4D, the HR estimate for canagliflozin vs. a non-SGLT2 inhibitor for HHF was 0.39 [76]. In a network meta-analysis of 23 studies, compared to DPP-4 inhibitors, SGLT2 inhibitors reduced the risk of HHF [77].

SGLT2 inhibitors (all four SGLT2 inhibitors) were able to reduce the risk of HF or HHF compared with control group (such as placebo or other antidiabetic drugs), regardless of cardiovascular disease. This view is supported by the combined results of clinical trials and meta-analyses.

#### Cardiovascular death

The beneficial effect of SGLT2 inhibitors on cardiovascular death now appears indisputable, whether meta-analyses, retrospective studies, or large clinical trials (Table 3) showed similar data that SGLT2 inhibitors lowered the risk of cardiovascular death. All four SGLT2 inhibitors are able to achieve this and are superior to DPP-4 inhibitors [12, 54, 55, 60, 75, 77]. In CANVAS, canagliflozin was slightly related to a lower risk of cardiovascular death compared with placebo (HR = 0.87) [78]. In EMPA-REG OUTCOME, the relative risk of cardiovascular death was decreased by 38% in the empagliflozin group [69]. Interestingly, dapagliflozin in the DAPA-HF and DECLARE–TIMI 58 studies achieved different cardiovascular mortality outcomes [13, 79]. Ertugliflozin does not appear to significantly reduce cardiovascular death compared with placebo (HR = 0.92) [11]. Outcomes from a large meta-analysis showed that SGLT2 inhibitors prevented cardiovascular death (P < 0.0001). Evidence that individual agents had different effects on cardiovascular outcomes or death was not evident (all I^2^ < 43%) [60]. SGLT-2 inhibitors obviously decreased cardiovascular death compared to DPP-4 inhibitors (RR = 0.88), in contrast to GLP-1 receptor agonists, there was no difference [77].

#### Arrhythmia

Several meta-analyses have showed that SGLT2 inhibitors decreased the risk of atrial fibrillation [58, 80]. A meta-analysis of 22 trials reported SGLT2 inhibitors were related to a lower risk of atrial fibrillation (RR = 0.82), atrial fibrillation/flutter (RR = 0.82), and ventricular tachycardia (RR = 0.73). The risk reduction for atrial flutter (RR = 0.83) and cardiac arrest (RR = 0.83) did not reach statistical significance [58]. Another meta-analysis indicated that the incidence of atrial fibrillation was 0.9% in subjects who received SGLT2 inhibitors and 1.1% in subjects who received placebo. The incidence of atrial fibrillation was significantly reduced (RR = 0.79) in both T2DM and non-T2DM patients receiving SGLT2 inhibitors [80].

Although current evidence supports that SGLT2 inhibitors reduce the risk of ventricular tachycardia, and atrial fibrillation in patients with or without T2DM, this is limited to meta-analyses, while the mechanisms are not well elucidated. More clinical trials are required to support the benefit of SGLT2 inhibitors in cardiac arrhythmias.

#### Nonfatal myocardial infarction

Although several meta-analyses have reported reductions in non-fatal myocardial infarction with SGLT-2 inhibitors, most have shown no apparent effect [55, 59, 60, 75]. Data from representative clinical trials (Table 3) showed that SGLT-2 inhibitors did not notably reduce the risk of non-fatal myocardial infarction. In CANVAS only, canagliflozin reduced the risk of non-fatal myocardial infarction to some extent compared with placebo (HR = 0.85) [78]. When compared with GLP-1 receptor agonists, the risk of non-fatal myocardial infarction was similar for both [77]. Based on the results of the current clinical trials and meta-analyses, it can be speculated that the effect of SGLT2 inhibitors on non-fatal myocardial infarction may be neutral. Potent evidence is now needed to support this.

#### Stroke

Results from some randomized controlled trials indicated that only canagliflozin among SGLT-2 inhibitors (Table 3) decreased the risk of stroke to a certain extent. In the CANVAS, canagliflozin had a similar effect to placebo on fatal or non-fatal stroke in patients with (HR = 0.84) and patients without a history of HF (HR = 0.88) [72]. In EMPA-REG OUTCOME, empagliflozin slightly increased the risk of fatal or non-fatal stroke (HR = 1.18; P = 0.26), and only for non-fatal stroke (HR = 1.24) [69].

Large meta-analyses displayed that SGLT-2 inhibitors barely reduced the risk of non-fatal stroke and even increased the risk (relative risk 1.30; P = 0.049) [55, 60]. This was also the case in a large retrospective study showing that SGLT2 inhibitors were less beneficial for ischemic stroke (HR = 0.85) [54]. Notably, a meta-analysis reported that SGLT2 was related to a lower risk of embolic stroke (RR = 0.32) [58]. Interestingly, the risk of non-fatal stroke was similar between GLP-1 receptor agonists and SGLT2 inhibitors, while only GLP-1 receptor agonists decreased the risk of non-fatal stroke when compared to placebo [77].

CKD with decreased estimated glomerular filtration rate (eGFR) or increased proteinuria increases the risk of hemorrhagic and ischemic stroke [81]. Outcomes from the CREDENCE trial and meta-analysis showed that 142 patients had a stroke (HR = 0.77). Effects on stroke subtypes were: ischemic (HR = 0.88), hemorrhagic (HR = 0.50), and indeterminate (HR = 0.54) [81]. There was evidence that the effect of SGLT2 inhibitors on total stroke varies by baseline eGFR (P = 0.01), with protection in the lowest eGFR (< 45 mL/min/1.73 m^2^) subgroup (HR = 0.50) [81].

SGLT2 inhibitors generally do not increase the risk of non-fatal stroke, and different results are reflected between different SGLT2 inhibitors, such as canagliflozin and empagliflozin. Several meta-analyses have also shown that SGLT2 inhibitors may lower the risk of certain types of stroke, such as embolic stroke. SGLT2 inhibitors on stroke risk may vary in different populations, such as depending on the level of renal function.

### Renal protection

Diabetes is the most common cause of CKD, accounting for approximately 50% renal failure cases requiring replacement therapy [82]. DKD develops in 30–50% of diabetic patients [83]. SGLT-2 inhibitors have been clearly shown in multiple studies to improve renal outcomes in patients with CKD or DKD (Table 4), and glomerular hemodynamic function, significantly reduces the risk of proteinuria and renal failure [5, 78, 82, 84–86].

**Table 4:** Associated renoprotective effects of SGLT2 inhibitors, results from randomized controlled trials

Study	Drugs	Composite outcome^a^	Progression of albuminuria	dSCr	Renal death	ESKD	40% eGFR reduction
CANVAS Program^b^ [78, 85]	Canagliflozin	5.5	89.4	1.2		0.4	5.3
	Placebo	9.0	128.7	2.4		0.6	8.7
	P value	0.3868	0.0184				
CREDENCE [70]	Canagliflozin	11.1		5.4	0.1	5.3	3.5
	Placebo	15.5		8.5	0.2	7.5	5.7
	P value	0.00001		< 0.001		0.002	
DECLARE–TIMI 58 [93]	Dapagliflozin	1.5			0.1	0.1	1.4
	Placebo	2.6			0.1	0.2	2.5
	P value	< 0.0001			0.32	0.013	< 0.0001
DAPA-CKD [122]	Dapagliflozin	6.6			< 0.1	5.1	5.2
	Placebo	11.3			0.3	7.5	9.3
	P value	< 0.001					
EMPEROR-Reduced [68]	Empagliflozin	1.6					
	Placebo	3.1					
	P value						< 0.001
EMPA-REG OUTCOME [86]	Empagliflozin	1.7	11.2	1.5			
	Placebo	3.1	16.2	2.6			
	P value	< 0.001	< 0.001	< 0.001			
VERTIS CV [11]	Ertugliflozin	3.2		3.1	0		
	Placebo	3.9		3.8	0		

*dSCr *doubling of serum creatinine, *ESKD *end-stage kidney disease, *eGFR *estimated glomerular filtration rate

^a^Primary renal composite outcome

^b^Rate 1000 people per year

^c^P value for homogeneity between CANVAS and CANVAS-R

SGLT2 inhibition was related to an acute, dose-dependent decrease in eGFR of about 5 mL/min/1.73 m^2^ and a reduction in albuminuria of approximately 30–40%. These effects reflect preclinical observations showing that proximal tubular sodium excretion activates tubulo-glomerular feedback by increasing the delivery of sodium and chloride, subsequently causing afferent vasoconstriction [87]. Due to reduced glomerular filtration, CKD patients have attenuated glucosuria and weight loss (eGFR < 60 mL/min/1.73 m^2^) [87]. A potential mechanism for the renoprotective effect of SGLT2 inhibitors may also involve uric acid reduction [88, 89]. In T2DM patients with stage 2 or 3 CKD, SGLT2 inhibitors improved HbA1c and urinary albumin/creatinine ratios without increasing serious adverse renal events [90, 91].

The National Kidney Foundation convened a scientific symposium of an international panel of more than 80 experts to elucidate and support the role of SGLT2 inhibitors in T2DM and CKD [82]. The notion that the benefits of SGLT2 inhibitors are mediated by non-glycemic mechanisms at the meeting is supported by a number of observations that CKD and cardiovascular disease risk reductions in clinical trials of these drugs have not been associated with glycemic control or the use of other antidiabetic drugs [82].

Dapagliflozin and canagliflozin are approved for CKD or DKD. In the CANVAS program, canagliflozin was related to a lower incidence of the composite of sustained doubling of serum creatinine (dSCr), ESKD, and renal death compared with placebo (HR = 0.53) [78, 85]. Canagliflozin may be beneficial for the progression of albuminuria (HR = 0.73) and the composite outcome of sustained 40% decrease in eGFR, requirement for renal replacement therapy, or renal death (HR = 0.60) [78, 85]. In CREDENCE, the risk of renal failure was lower in the canagliflozin group compared with the placebo group [70]. Results from CANTATA-SU secondary analysis showed that compared with glimepiride, canagliflozin slowed the progression of renal disease over 2 years, and that it may confer renoprotective effects independent of its glycemic effects [92]. Dapagliflozin had a reduced incidence of renal events in the DECLARE–TIMI 58 trial (4.3% vs. 5.6%) [13]. The cardio-renal secondary composite outcome was obviously lower with dapagliflozin (P < 0.0001). The sustained decline in eGFR was reduced by 46% (P < 0.0001). Compared with placebo, dapagliflozin was associated with a lower risk of renal death or ESKD (0.1% vs. 0.3%; P = 0.012) [93]. A prespecified analysis from DAPA-CKD showed that when added to ACEI or ARB therapy, dapagliflozin decreased the risk of several clinical outcomes (such as ≥50% eGFR decline and ESKD) in patients with CKD [94].

Empagliflozin demonstrated renal protection in T2DM patients, as demonstrated in EMPA-REG OUTCOME, where empagliflozin was related to slower renal disease progression and a lower incidence of clinically relevant renal events compared with placebo (Table 4) [86]. Additionally, compared with placebo, empagliflozin improved uric acid levels with a lower risk of serious renal outcomes [68, 86, 88]. A sub-analysis of EMBODY trial reported that empagliflozin prevented the decline of renal function in T2DM patients with acute myocardial infarction, especially those with a baseline eGFR ≥ 60 mL/min/1.73 m^2^ [88]. Uric acid reduced about 0.9 mg/dL in the empagliflozin group (P < 0.001) [88].

SGLT-2 inhibitors decrease renal failure risk in multiple meta-analyses [12, 55–57]. SGLT2 inhibitors reduced the risk of kidney disease progression by 45% (P < 0.0001), including in some patients, regardless of atherosclerotic cardiovascular disease. The magnitude of the benefit of SGLT2 inhibitors varies with baseline renal function [61]. A meta-analysis including 32,949 patients showed that SGLT-2 inhibitors reduced the risk of renal events (RR = 0.68) [57]. Compared with GLP-1, SGLT-2 inhibitors were related to a lower risk of renal events (RR = 0.79) [57]. Moreover, dapagliflozin (HR = 0.62, 0.60, 0.68 and 0.63) and empagliflozin (HR = 0.64, 0.61, 0.69 and 0.64) notably decreased renal function progression compared with duraglutide, exenatide, liraglutide, and lixisenatide [56].

### All-cause mortality

Results from several SGLT-2 inhibitor cardiovascular outcomes investigation trials indicated that SGLT-2 inhibitors decreased the risk of all-cause mortality compared with placebo. In the CANVAS program, compared with placebo, canagliflozin displayed a greater benefit on all-cause mortality in patients with a history of HF (HR = 0.70) than in patients without HF history (HR = 0.93) [72]. In EMPA-REG OUTCOME, empagliflozin reduced the risk of all-cause mortality by 32% (P < 0.001) [69]. In contrast, dapagliflozin did not notably lower the risk of all-cause mortality compared with placebo in DECLARE–TIMI 58 (HR = 0.93) [13]. In DAPA-HF, dapagliflozin reduced cardiovascular mortality to some extent (HR = 0.83) in subjects with HFrEF regardless of a previous history of diabetes [79]. There was a similar all-cause mortality between ertugliflozin and placebo (HR = 0.93) [11].

Several meta-analyses have showed that SGLT-2 inhibitors lower the risk of all-cause mortality [12, 55, 59, 60, 95]. A network meta-analysis of 236 trials showed that SGLT-2 inhibitors (HR = 0.80) were obviously reduced all-cause mortality than controls. SGLT-2 inhibitors (HR = 0.78) were associated with lower mortality compared to DPP-4 inhibitors [75]. SGLT-2 inhibitors reduced deaths by 5–48 per 1000 patients over 5 years [55].

Overall, all SGLT2 inhibitors, except ertugliflozin, were able to significantly reduce all-cause mortality, and the corresponding meta-analyses showed results consistent with clinical trials.

### Anemia

SGLT-2 inhibitors also reduce hepcidin levels, improve erythropoiesis, increase hemoglobin levels, and reduce the incidence of anemia [28, 96–98]. A post hoc analysis from CREDENCE displayed that 13% of the 4401 participants developed anemia or started treatment for anemia. Mean hemoglobin concentrations were 7.1 g/L higher and hematocrit was 2.4% higher in canagliflozin compared to placebo. The risk of the composite outcome of anemia or initiation of anemia treatment was lower in the canagliflozin group compared to the placebo group (HR = 0.6; P < 0.0001). Subjects received canagliflozin had a lower risk of anemia events (0.58; P < 0.0001) alone, initiation of iron preparations (0.64; P < 0.0001), and need for erythropoiesis-stimulating agents (0.65; P = 0.012) [96].

Results of a retrospective cohort study indicated that SGLT2 inhibitors usage was related to an obviously lower prevalence of anemia [odds ratio (OR) = 0.35]. The adjusted MD for hemoglobin levels between SGLT2 inhibitor subjects and non-users was 7.0 g/L [97]. A corresponding meta-analysis reported that SGLT2 inhibitors significantly increased hemoglobin levels compared with placebo (P < 0.00001), and each SGLT2 inhibitor resulted in a notable increase in hematocrit levels (P < 0.00001) [98].

## T2DM, cardiovascular and renal outcomes associated with SGLT2 inhibitors

### Canagliflozin

Multiple clinical trials have shown that canagliflozin significantly reduces glycemic compared to placebo (Table 2). Canagliflozin showed non-inferiority compared to the active control group, especially canagliflozin 300 mg showed superiority and reduced FPG and SBP [99–101]. In the CALMER study, SGLT2 inhibitor combined with DPP-4 inhibitor significantly decreased glycemic variability compared with monotherapy [102].

FDA approval of canagliflozin for reducing the risk of heart attack, stroke or cardiovascular death in adults with T2DM and established cardiovascular disease was based on a consensus report from the ADA/European Association for the Study of Diabetes, these reports supported the use of canagliflozin in a broad range of patients. For T2DM patients with cardiovascular disease, the ADA recommends pharmacological management with SGLT2 inhibitors, which in particular have proven cardiovascular benefits [16, 103, 104]. The AACE also stated that canagliflozin has been shown to reduce MACE in suitable patients [16, 104].

The CANVAS program combined data from CANVAS and CANVAS-R, recruited 10,142 T2DM subjects with high cardiovascular risk [78, 85]. The CANVAS program aimed to evaluate the effect of canagliflozin on cardiovascular disease risk in patients with poorly controlled T2DM [105]. As a result, the incidence of the primary outcome was lower with canagliflozin compared to placebo [78]. HHF or cardiovascular death was reduced in the canagliflozin-treated group compared to placebo (HR = 0.78) [72].

Canagliflozin is FDA-approved to treat DKD and reduce the risk of HHF in T2DM patients with DKD based on CREDENCE. CREDENCE is the first renal outcome study to specifically address any SGLT2 inhibitor in addition to standard of care in T2DM patients with DKD [16]. However, CREDENCE was discontinued early following a planned interim analysis based on the recommendations of the data and safety monitoring board [70]. Canagliflozin reduced the risk of the primary outcome by 30% (P = 0.00001) [70]. The relative risk of the renal-specific composite risk of ESKD, dSCr, or renal death was decreased by 34%, and the relative risk of ESKD was reduced by 32% (P = 0.002) [70].

### Empagliflozin

Among the currently used SGLT2 inhibitors, empagliflozin has the highest SGLT2 specificity. The low risk of hypoglycemia and weight loss properties of empagliflozin and its cardiovascular benefits support its use as a first-line alternative to metformin [106, 107].

Multiple randomized controlled trials demonstrated that compared with placebo, empagliflozin provided reducing in HbA1c, FPG and SBP, and weight loss (Table 2). The hypoglycemic efficacy of empagliflozin was noninferior to sitagliptin or glimepiride. Empagliflozin reduced SBP and body weight compared with sitagliptin. Empagliflozin 25 mg was better than sitagliptin in decreasing HbA1c [108–113].

Several randomized controlled trials have demonstrated the efficacy of empagliflozin as an add-on in patients with T2DM [114, 115]. One 104-week phase 3 trial compared the efficacy of empagliflozin and glimepiride as an add-on to metformin in T2DM [114]. The result was that empagliflozin was noninferior to glimepiride during treatment. The adjusted MD for change from baseline in HbA1c was − 0.11% [114].

The US FDA approved empagliflozin for reducing cardiovascular death in T2DM adults due to the wonderful outcomes in EMPA-REG OUTCOME [15, 69]. The primary composite outcome was nonfatal myocardial infarction, cardiovascular death, or nonfatal stroke. The primary outcome reported in 10.5% and 12.1% of patients in the empagliflozin group and the placebo group (HR = 0.86; P = 0.04), respectively. Cardiovascular death and HHF were obviously decreased in the empagliflozin group [69]. No significant between-group difference in the key secondary outcome (P = 0.08) [69]. Similar outcomes were shown in elderly patients [116].

Two randomized controlled trials have demonstrated empagliflozin for adults with HFrEF [62, 68]. FDA approval of empagliflozin in adults with HFrEF was based on the EMPEROR-Reduced trial [68]. The primary outcome was a composite of cardiovascular death or hospitalization for worsening HF. The primary outcome events reported in 19.4% and 24.7% of the empagliflozin group and the placebo group (HR = 0.75; P < 0.001), respectively. The effect of empagliflozin on the primary outcome of patients was consistent regardless of T2DM [68].

Recently, empagliflozin was approved for the treatment of HF with preserved ejection fraction in adults. The approval was based on outcomes from the landmark EMPEROR-Preserved trial [117]. The primary outcome of this trial was a composite of cardiovascular death or HHF, those reported in 13.8% and 17.1% of subjects in the empagliflozin group and the placebo group within a median of 26.2 months (HR = 0.79; P < 0.001), respectively. Empagliflozin exerted similar effects in T2DM or non-T2DM patients. The total number of HHF was lower in the empagliflozin group compared to placebo (P < 0.001) [117].

The long-term effect of empagliflozin on the kidneys was determined by investigators in the EMPA-REG OUTCOME and EMPEROR-Reduced trials [68, 86]. The incidence of kidney disease or worsening kidney disease was 12.7% in the empagliflozin group and 18.8% in the placebo group (HR = 0.61; P < 0.001). A dSCr in the empagliflozin group had an obviously lower relative risk of 44%. The relative risk of renal replacement therapy was reduced by 55% in the empagliflozin group. No significant between-group difference in the incidence of albuminuria [86]. Empire HF Renal was a prespecified sub-study of the investigator-initiated Empire HF trial to investigate the effect of empagliflozin on GFR, plasma volume, and extracellular volume in patients with HFrEF [118]. Empagliflozin therapy led to a reduction in estimated extracellular volume (adjusted MD − 0.12 L; P = 0.00056), estimated plasma volume (− 7.3%; P < 0.0001), and measured GFR (− 7.5 mL/min; P = 0.00010) compared to placebo [118].

### Dapagliflozin

The hypoglycemic effect of dapagliflozin in randomized controlled trials was similar to that of previous SGLT-2 inhibitors (Table 2) [89, 119, 120]. Add-on therapy with dapagliflozin offered additional clinical benefits for patients with poorly controlled T2DM with sitagliptin or with or without metformin [120]. Compared with monotherapy, exenatide combined with dapagliflozin improved various glycemic parameters and cardiovascular risk factors in T2DM patients who were not adequately controlled by metformin [121]. Compared with pioglitazone, dapagliflozin showed non-inferiority in glycemic control [43]. Sitting SBP was notably lower in the dapagliflozin group compared with placebo (P = 0.0002) [89]. Its hypotensive properties are particularly beneficial in patients already receiving beta-blockers or calcium channel blockers [89].

The cardiovascular safety of dapagliflozin was established in the DECLARE–TIMI 58 trial. FDA approval of dapagliflozin for reducing the risk of HHF in patients with T2DM is based on this trial. The primary outcome of DECLARE–TIMI 58 was the composite of MACE. The primary efficacy outcomes were MACE and a composite of cardiovascular death or HHF [13]. Dapagliflozin met the prespecified non-inferiority criteria for MACE (P < 0.001). Dapagliflozin did not decrease the incidence of MACE (HR = 0.93) compared with placebo, while resulted in a reduction in cardiovascular death or HHF (4.9% vs. 5.8%; P = 0.005) [13].

Based on positive outcomes from the landmark DAPA-HF trial, the FDA approved dapagliflozin for cardiovascular death and HHF in patients with HFrEF [71]. Dapagliflozin achieved a statistically and clinically meaningful decrease in cardiovascular death or HHF compared with placebo [18, 71, 79]. The primary outcome was a composite of worsening HF or cardiovascular mortality and reported in 16.3% and 21.2% of patients in the dapagliflozin group and the placebo group (P < 0.001), respectively. A first worsening HF event occurred in 10.0% of patients in the dapagliflozin group and in 13.7% in the placebo group (HR = 0.70) [79]. Among patients without diabetes and with HbA1c levels below 5.7%, the primary outcome rate was 12.1% in dapagliflozin and 16.9% in placebo. Among patients with HbA1c ≥ 5.7%, the incidence of the primary outcome was 13.7% in dapagliflozin and 18.0% in placebo (HR = 0.74) [71].

FDA approval of dapagliflozin to teart CKD patients with or without risk of T2DM progression was based on positive results from the DAPA-CKD trial [122]. An independent data monitoring committee recommended stopping the trial because of efficacy. The incidence of the primary outcome event was 14.5% in the placebo group and 9.2% in the dapagliflozin group (P < 0.001). The HR for the composite of sustained at least 50% decline in eGFR, ESKD or death from renal causes was 0.56 (P < 0.001), and was 0.71 for death from cardiovascular causes or HHF (P = 0.009) [122]. 4.7% of patients in the dapagliflozin group and 6.8% of the placebo group (P = 0.004) occurred death [122].

### Ertugliflozin

Ertugliflozin received its first FDA approval for T2DM on December 2017, based on positive outcomes from multiple randomized controlled trials. These trials demonstrated that ertugliflozin, alone or as an add-on, was superior to placebo in glycemic control in T2DM as a combination in patients receiving metformin and sitagliptin, provided reductions in HbA1c and FPG, SBP and body weight, with good tolerability. Ertugliflozin 5 mg has similar effects to ertugliflozin 15 mg, sometimes ertugliflozin 5 mg may be even more effective (Table 2) [123–127]. Treatment with ertugliflozin led to greater reductions in SBP or body weight compared to glimepiride, sitagliptin, or placebo.

The multicenter, double-blind trial VERTIS CV determined the effect of ertugliflozin on cardiovascular outcomes (Table 3) [11]. Cardiovascular death or HHF reported in 8.1% and 9.1% patients in the ertugliflozin group and the placebo group (P = 0.11), respectively. The HR for renal death, renal replacement therapy, or dSCr was 0.81 [11]. Ertugliflozin did not notably decrease first HHF or cardiovascular death (HR = 0.88). The risk reduction was greater with an ejection fraction ≤ 45% (HR = 0.48) versus an ejection fraction > 45% (HR = 0.86). Ertugliflozin decreased the risk of total HHF (RR = 0.70) and HHF/cardiovascular death (RR = 0.83) [74]. Ertugliflozin led to reducing in SBP, HbA1c, and body weight, maintained eGFR in patients with CKD stage 3A. Results were broadly similar in the CKD stage 3B subgroup, except for the diminished HbA1c response to ertugliflozin 15 mg [128].

## Safety and tolerability

Several clinical trials involving SGLT-2 inhibitors in T2DM patients have shown more adverse reactions such as genital infections, amputations, DKA, and fractures in SGLT-2 inhibitor compared with placebo. There is little or no evidence of effects of SGLT-2 inhibitors on blindness, amputation, neuropathic pain, eye disease, or health-related quality of life in large meta-analyses [55, 129]. Compared with GLP-1 agonists, SGLT-2 inhibitors were related to a lower risk of adverse events resulted in patient withdrawal [75]. SGLT2 inhibitors significantly decreased the risk of all serious adverse events (HR = 0.91, P < 0.001) and acute kidney injury (AKI) (HR = 0.74; P < 0.001) [14]. A meta-analysis reported dose-independent adverse events with SGLT2 inhibitors [130]. Compared with controls or placebo, SGLT2 inhibitors did not increase the risk of acute pancreatitis [131].

Overall adverse events and adverse event-related discontinuation rates were generally similar between placebo, sitagliptin, glimepiride, and SGLT-2 inhibitors [99, 100]. However, canagliflozin 100 mg had a higher incidence of adverse events than placebo or sitagliptin [100]. The safety profile of empagliflozin was similar to sitagliptin or metformin, and most adverse events were mild or moderate in intensity [110]. Adverse events were similar between dapagliflozin and placebo, with few adverse events associated with volume depletion (< 1% vs. 0%) or renal function (1% vs < 1%) [89]. The most common adverse events related to dapagliflozin in DURATION-8 were diarrhea, injection site nodules, nausea, and urinary tract infection [121]. In DAPA-HF, the incidence of adverse reactions associated with volume depletion, renal insufficiency, as well as hypoglycemia did not differ between placebo and dapagliflozin [79]. The incidence of hypovolemia, symptomatic hypoglycemia, and urinary tract infection did not differ obviously between groups in patients receiving ertugliflozin compared with placebo or metformin [124, 125].

### Genital infection or urinary tract infection

Both retrospective studies and meta-analyses have indicated that SGLT-2 inhibitors cause genital infections at a higher risk than other antidiabetic drugs [34, 53, 55, 129]. Safety analysis indicated a continued increase in the risk of genital infections associated with SGLT-2 inhibitors (relative risk, regulatory submission 4.75; Scientific Reports 2.88) [60].

Genital fungal infections adverse events have been associated with higher rates of canagliflozin, with a minority leading to treatment discontinuation [99, 100]. Empagliflozin has a higher risk of genital infection or urinary tract infection than placebo, metformin, or sitagliptin [109, 110, 117]. Increased rates of genital infections were more commonly reported in patients receiving empagliflozin in EMPA-REG OUTCOME and EMPEROR-Reduced trials [68, 69]. Common adverse reactions commonly reported with dapagliflozin in DURATION-8 included urinary tract infections [121]. Genital infections leading to treatment discontinuation or considered serious adverse events in DECLARE–TIMI 58 were more common in the dapagliflozin group (0.9% vs. 0.1%, P < 0.001) [13]. In VERTIS MONO, the incidence of female genital fungal infections was notably higher in the ertugliflozin group than in the placebo or metformin group. In men, the 15 mg group was significantly higher compared with placebo/metformin [124]. In VERTIS SITA2, compared with placebo (0–1.9%), female and male subjects receiving ertugliflozin (3.7–14.1%) had a higher incidence of genital fungal infections [125].

### Osmotic diuresis

In CANTATA-SU, canagliflozin was related to a higher risk of osmotic diuresis-related events compared with glimepiride [99]. A meta-analysis reported that for osmotic diuresis, SGLT2 inhibitors notably increased the risk by 75% (P = 0.036). Subgroup analysis clarified that this effect was entirely attributable to the increase in patients with eGFR ≥ 60 ml/min/1.73 m^2^ [14].

### Hypoglycemia

Meta-analysis results showed that the risk of hypoglycemia with SGLT-2 inhibitors was similar to that of other drugs [34, 124]. SGLT-2 inhibitors have a low hypoglycemia risk and are noninferior to placebo when used as monotherapy or combination therapy [8, 109, 119, 124].

Canagliflozin has a higher incidence of hypoglycemia compared with placebo or sitagliptin [100]. Compared with placebo, the incidence of symptomatic hypoglycemia was higher with ertugliflozin 15 mg [123]. Empagliflozin was related to a lower risk of hypoglycemia compared with glimepiride (2% vs. 24%, P < 0.0001) [114]. Hypoglycemic events associated with dapagliflozin were rare and not serious [119].

### Sarcopenia

The mechanism of fat mass reduction with SGLT2 inhibitors is thought to be the result of lipolysis in adipose tissue due to activation of gluconeogenesis. However, activation of the gluconeogenesis system induces not only lipolysis in adipose tissue but also proteolysis in skeletal muscle, which supplies amino acids as substrates to the liver, thus leading to sarcopenia [132]. Weight loss and sarcopenia have also been reported in case reports with SGLT2 inhibitors [133].

### Cancer

Data from a large meta-analysis suggested that dapagliflozin was related to an unbalanced incidence of breast and bladder cancer compared with controls [34]. However, two meta-analyses achieved similar outcomes [134, 135]. Results of a meta-analysis reported that SGLT2 inhibitors were not notably related to increased cancer risk compared with other antidiabetic drugs or placebo (OR = 1.14). For prespecified cancer types, use of SGLT2 inhibitors (OR = 3.87), especially empagliflozin (OR = 4.49), would increase bladder cancer incidence. Canagliflozin may be protective against gastrointestinal cancer (OR = 0.15) [135].

Of note, the EMPA-REG OUTCOME trial accounted for more than half of the individuals and events in the entire analysis [69, 135, 136]. In the intention-to-treat analysis, bladder cancer was reported in 0.2%, 0.1%, and 0.4% of patients in the placebo, empagliflozin 10 mg, and empagliflozin 25 mg groups, respectively [136]. When exposure time (6 months) was considered, the incidence was 0.2%, 0.1% and 0.3%, respectively. Overall, no evidence of an imbalance was observed in bladder cancer cases between empagliflozin and placebo [136].

### Ketoacidosis

On December 4, 2015, a safety review by the US FDA resulted in the addition of a warning to the label of SGLT2 inhibitors, stating that there was too much acid in the blood [137]. On March 19, 2020, the FDA approved changes to the prescribing information for SGLT2 inhibitor diabetes medications to reduce the risk of DKA after surgery, recommending temporary discontinuation of these medications prior to scheduled surgery [137]. Normoglycemic DKA secondary to SGLT2 inhibitors in T2DM is a rare but increasingly reported phenomenon [138]. In DECLARE–TIMI 58, the incidence of DKA was higher in the dapagliflozin group than in the placebo group (0.3% vs. 0.1%, P = 0.02) [13]. In one cohort study, the incidence of DKA was 0.43 percent [138]. Normoglycemic DKA was most common in patients taking canagliflozin, followed by empagliflozin and dapagliflozin. Infection (32.6%) was the most common trigger for DKA, followed by insulin nonadherence (13.7%). Canagliflozin was most potently associated with the development of euglycemic DKA and was associated with the highest rate of medical intensive care unit admissions (66.6%) [138].

Several meta-analyses have indicated that SGLT2 inhibitors increased the risk of DKA [139, 140]. A meta-analysis indicated that SGLT2 inhibitors were statistically related to an increased risk of DKA compared with controls (0.18% vs. 0.09%, OR = 2.13) [140]. In contrast, another meta-analysis of 109 trials reported that SGLT2 inhibitors did not increase the risk of DKA (RR = 0.66) compared with placebo [129].

The above results concluded that SGLT2 inhibitors can increase the risk of DKA, despite a meta-analysis reported that SGLT2 inhibitors did not increase that. Additional evidence is two possible mechanisms have been proposed: free fatty acid production followed by conversion to ketone bodies; SGLT2 inhibitors stimulate glucagon secretion leading to the production of ketone bodies [129].

### Fracture

The U.S. FDA strengthened the warning that canagliflozin was associated with an increased risk of fractures and added novel information about reducing bone mineral density [141].

Fracture risk assessment in CANVAS program in patients with T2DM and a history of cardiovascular disease who received canagliflozin or placebo showed 496 subjects who recorded ≥ 1 fracture event. There was obvious heterogeneity in the effects of CANVAS (HR = 1.55) and CANVAS-R (HR = 0.86) on fracture (P = 0.005) [142]. However, the fracture rate of canagliflozin was not significantly different from placebo in CREDENCE [70].

In recent years, multiple meta-analyses have reported no increased fracture risk with SGLT2 inhibitors compared with placebo [129, 143–145]. Canagliflozin was not related to an increased risk of fracture compared with GLP-1 agonists [146].

Canagliflozin was not included, although SGLT2 inhibitors did not increase fracture risk, according to the results of the meta-analysis. Users still need to heed the warnings from the FDA.

### Amputation

In the CANVAS program, the risk of amputation associated with canagliflozin (HR = 1.97) was increased, with amputations mainly at the toe or metatarsal [78]. In VERTIS CV, 2.0% of patients receiving ertugliflozin 5 mg and 2.1% receiving ertugliflozin 15 mg underwent amputation compared with 1.6% receiving placebo [11]. Additionally, a large meta-analysis reported that canagliflozin increased the risk of amputation [12]. A meta-analysis of 14 trials indicated that SGLT2 inhibitors were not significantly related to an increased risk of diabetic foot syndrome compared with placebo (OR = 1.05). Amputation rates were increased in patients with T2DM who received canagliflozin but not empagliflozin [147]. Patients with atherosclerotic cardiovascular disease had a higher risk of amputation than those without atherosclerotic cardiovascular disease (RR 1.44 vs. 0.96, P = 0.066) [139].

There was no difference in amputation rates for canagliflozin in CREDENCE [70]. In OBSERVE-4D, the HR estimates for canagliflozin vs. no SGLT2 inhibitor for lower-knee and lower extremity amputations were 0.75 and 1.01 in an intention-to-treat analysis [76].

There were no obvious differences in amputation events by type of SGLT2 inhibitor, by baseline population, and by duration of SGLT2 inhibitor use [148].

There may be a risk of amputation following the use of SGLT2 inhibitors, usually in the toe or metatarsal, and limited to canagliflozin.

### Acute kidney injury

Although SGLT2 inhibitors stabilize eGFR and reduce renal function progression and uric acid, the US FDA has strengthened existing warnings about the risk of AKI with canagliflozin and dapagliflozin and recommended to assess renal function before initiating canagliflozin or dapagliflozin, followed by periodic monitoring. In the event of AKI, the drug should be discontinued immediately and renal impairment treated [149].

In clinical studies, SGLT2 inhibitors had the lowest risk of AKI (5.59%) compared with other drugs and controls [150]. Several recent meta-analyses indicated that SGLT2 inhibitors can reduce the risk of AKI [151, 152]. A network meta-analysis of 18 studies including 156,690 patients with T2DM reported that SGLT2 inhibitors were related to a lower risk of AKI compared with placebo (OR = 0.76). Furthermore, SGLT2 inhibitors were significantly related to a reduced risk of AKI compared with GLP-1 receptor agonists (OR = 0.79) and DPP-4 inhibitors (OR = 0.68) [151].

A retrospective study showed AKI in 0.3% of patients receiving SGLT2 inhibitors. After adjustment, patients who developed AKI were more likely to be male, were ≥ 65 years old, had a lower body mass index, had a history of HF, and used diuretics more frequently than those who did not [153].

Both clinical studies and meta-analyses have shown that SGLT2 inhibitors are related to lower AKI, but patients are still at risk of developing AKI. There are three mechanisms by which SGLT2 inhibitors may increase the risk of AKI: decreased effective volume due to excessive diuresis; excessive drop in transglomerular pressure; induces renal medulla hypoxic injury, associated with enhanced distal tubular transport [129, 154].

## SGLT2 inhibitor pharmacogenomics

SGLT2 is encoded by the *SLC5A2* gene, which is located on chromosome 16 [155]. Since SGLTs reabsorbs approximately 90% of filtered glucose, *SLC5A2* mutations may affect SGLT2 expression levels to affect the proportion of glucose reabsorption, resulting in familial renal glycosuria, which is characterized by isolated glucosuria without overt hyperglycemia [155]. Common genetic variants in *SLC5A2* are associated with increased AUC120min_glucose_, increased AUC_glucose_ and altered 30 min glucose levels in subjects with impaired glucose tolerance and impaired fasting glucose [155]. Currently, for SGLT2 inhibitors, studies have focused on genes that affect renal glucose reabsorption (e.g., *SLC5A2*), but have not found an association between *SLC5A2* variants and response to empagliflozin [156]. Variations in the gene encoding SGLT2 are associated with the risk of generalized or sudden-onset HF. This association is mediated by distinct metabolic and physiological changes independent of the presence of T2DM or previous cardiovascular disease events [157].

Results from a meta-analysis showed that the rs9934336 G allele was nominally associated with increased 30-min plasma glucose, 120-min insulin concentration, and AUC120min_glucose_ (P < 0.05) [158]. Another prospective study showed that variant rs9934336 was significantly associated with decreased HbA1c in T2DM patients (P = 0.023). rs9934336 was significantly negatively correlated with the presence of T2DM (P < 0.05) [159]. The polymorphisms rs3813008 and rs3116150 were neither associated with glycemic parameters nor with T2DM [159]. Common genetic variants in the *SLC5A2* gene neither affect diabetes-related metabolic profiles nor response to SGLT2 inhibitor therapy [160]. Additionally, in a randomized controlled trial, weight loss with dapagliflozin plus exenatide was associated with the variant rs10010131 A allele [161].

SGLT2 inhibitors are metabolized in vivo by the uridine 5'-diphosphate glucuronyltransferase (UGT) isoform UGT1A9. In vitro studies have shown that *UGT1A9* gene variants lead to altered UGT enzyme activity. Therefore, variations in the UGT gene may affect the pharmacokinetics of SGLT2 inhibitors [156, 162]. Plasma canagliflozin exposure (C_max,ss_, 11%; AUC_τ,ss_, 45%) was increased in UGT1A9*3 carriers relative to wild type. Increased plasma canagliflozin exposure was observed in participants with the UGT2B4*2 genotype compared with UGT2B4*2 non-carriers (C_max,ss_, 21%; AUC_t,ss_, 18%) [162]. Subjects with the UGT1A9*3 allele had a 26% higher exposure to canagliflozin than those without the allele [163]. Ertugliflozin is primarily metabolized by glucuronidation of UGT1A9. A meta-analysis showed that the largest changes in ertugliflozin AUC occurred in subjects carrying the UGT1A9*3 heterozygous variant. *UGT1A9* genotype did not have any clinically meaningful effect on ertugliflozin exposure in healthy subjects [164].

A missense variant (I148M) in patatin-like phospholipase domain–containing protein 3 (PNPLA3) causes susceptibility to fatty liver disease [165]. PNPLA3 is expressed in liver and adipose tissue and mediates triacylglycerol hydrolysis [156, 166]. In the EFFECT-II trial, only dapagliflozin in combination with omega-3 (n-3) carboxylic acid reduced PDFF (P = 0.046) and total liver fat volume (relative -24%, P = 0.037) compared with placebo. There was an interaction between the *PNPLA3* I148M polymorphism and changes in hepatic PDFF in the active treatment group (P = 0.03) [166].

## Conclusions

In conclusion, SGLT2 inhibitors could manage uncontrolled glycemic in patients with T2DM who have received prior metformin, with a safety profile similar to placebo or other hypoglycemic agents (e.g., sitagliptin). SGLT2 inhibitors do not affect insulin secretion but have been clinically shown to improve β-cell function and improve insulin sensitivity. The effect of SGLT2 inhibitor on blood lipids is manifested as the increase of LDL and HDL, and the decrease of triglyceride. Compared with pioglitazone, SGLT2 inhibitors significantly reduced the fatty liver index, which means that SGLT2 inhibitors can be preferred drugs for T2DM patients with NAFLD. The cardiovascular benefits (e.g., HHF and cardiovascular death) of SGLT2 inhibitors are diverse, and the mechanisms are partially elucidated. However, the effect of SGLT-2 inhibitors on the risk of non-fatal stroke or myocardial infarction was neutral. SGLT2 inhibitors have been clearly shown in multiple trials to improve renal outcomes in patients with CKD or DKD, improve glomerular hemodynamic function, and significantly decrease the risk of proteinuria and renal failure. Although empagliflozin is not currently approved for this use, the results of the EMPA-REG OUTCOME and EMPEROR-Reduced trials are positive. SGLT2 inhibitors were also able to reduce all-cause mortality. SGLT2 inhibitors increased hemoglobin levels and prevented the occurrence of anemia events. To date, information on the role of genetic polymorphisms in response to SGLT-2 inhibitors is rather limited, and the mechanisms underlying the effects require more studies to reveal.

Genital or urinary tract infections, amputations, fractures (canagliflozin), osmotic diuresis, ketoacidosis, sarcopenia and hypotension are common with SGLT2 inhibitors. SGLT2 inhibitors have a lower risk of hypoglycemia than glimepiride. The FDA has also issued a safety warning for canagliflozin and dapagliflozin, which may cause AKI or fractures. The balance of benefits and risks and risk mitigation strategies for SGLT2 inhibitors should be carefully considered [82].

## Data Availability

Not applicable.

## Abbreviations

T2DM: Type 2 diabetes mellitus
SGLT2: Sodium-glucose cotransporter 2
EGP: Endogenous glucose production
HF: Heart failure
DKD: Diabetic kidney disease
ESKD: End-stage kidney disease
ADA: American Diabetes Association
AACE: American Association of Clinical Endocrinologists
HbA1c: Glycated hemoglobin
GLP1: Glucagon-like peptide 1
DKA: Diabetic ketoacidosis
CKD: Chronic kidney disease
HFrEF: HF with reduced ejection fraction
HHF: Hospitalization for heart failure
MD: Mean difference
SBP: Systolic blood pressure
RR: Ratio risk
OR: Odds ratio
HR: Hazard ratio
NAFLD: Nonalcoholic fatty liver disease
PDFF: Proton density fat fraction
ALT: Alanine aminotransferase
MACE: Major adverse cardiovascular events
FPG: Fasting plasma glucose
NT-proBNP: N-terminal pre-B-type natriuretic peptide
eGFR: Estimated glomerular filtration rate
dSCr: Doubling of serum creatinine
AKI: Acute kidney injury
UGT: Uridine 5ʹ-diphosphate glucuronyltransferase
PNPLA3: Patatin-like phospholipase domain–containing protein 3
